# The evaluation of muscle strength and architecture in type 1 diabetes mellitus: a cross-sectional study

**DOI:** 10.1186/s12902-022-01062-y

**Published:** 2022-06-07

**Authors:** Sefa Tan, Zafer Gunendi, Jale Meray, İlhan Yetkin

**Affiliations:** 1 Department of Physical Medicine and Rehabilitation, Polatli Duatepe State Hospital, Ankara, Turkey; 2 Department of Physical Medicine and Rehabilitation, Gazi University Faculty of Medicine, Ankara, Turkey; 3 Department of Endocrinology, Gazi University Faculty of Medicine, Ankara, Turkey

**Keywords:** Type 1 diabetes mellitus, Exercises, Ultrasonography, Muscle architecture

## Abstract

**Background:**

The aim of this study is to compare muscle strength and architecture between type 1 diabetes patients and healthy volunteers and to assess whether there is an ultrasonographic structural change in this population.

**Methods:**

Thirty-two patients with T1D (23 female, 9 male) with an age average of 31.3 ± 8.7 years, matched in terms of age, gender, height, weight and physical activity were included in the study. In the T1D and control group, ultrasonographic measurements of quadriceps femoris muscle (RF, VI, VM, VL) and pennate angle (VI, VM, VL) were performed. Muscle strength values were measured using isokinetic dynamometer system at angular velocities of 60º/s and 180º/s in both groups.

**Results:**

Initially, both groups were similar in demographic and clinical characteristics (*p* > 0.05). In the T1D group, there was a statistically significant difference in flexion/extension peak torque measurements at an angular velocity of 60º/s compared to the control group (*p* < 0.05). In support of these isokinetic measurements, RF, VI, VM, VL muscle thicknesses and VI, VM pennate angle measurements in T1Ds were significantly lower (*p* < 0.05). When the T1D group was subgrouped according to HbA1C and diabetes duration, there was no significant difference in ultrasonographic and isokinetic measurements between the two groups (*p* > 0.05). When the T1D group was subgrouped, in the group that used insulin pump RF, VI, VM muscle thickness measurements were significantly higher (*p* < 0.05) than the group using subcutaneous insulin.

**Conclusions:**

This study supports that muscle strength and architecture are adversely affected in the T1D patient group, insulin deficiency is a risk factor for sarcopenia and this can be shown through ultrasonography. It can also be said that insulin pump use has more positive effects in terms of diabetic myopathy than subcutaneous insulin, and diabetic myopathy develops independently of other diabetic complications. As a result, the muscle architecture of T1D people is adversely affected by insulin deprivation, so regular physical activity should be an integral part of diabetes treatment.

## Background

Type 1 diabetes mellitus (T1D) is a chronic, broad-spectrum metabolic disorder characterized by absolute insulin deficiency, progressing with complications and requiring continuous medical care [1]. The incidence rate is between 0.5–1%, and the incidence has reportedly increased globally by 3% year-on-year in the last decade [2]. In addition to the increase in the number of patients with T1D diagnosis, the age of diagnosis is also decreasing. This causes more frequent encounters with chronic complications of T1D [3].

While some of the diabetic complications are being kept at the very forefront, an important part is not in the foreground, as an example, complications of the musculoskeletal system can be mentioned [4]. Complications of the musculoskeletal system bring with them many risk factors for patients suffering from this disease and result in a decrease in the quality of life of the patients. Despite all this, unlike other well-known complications of T1D (such as nephropathy, retinopathy, neuropathy, cardiovascular disease), there is no standard guide for detecting and treating T1D-related musculoskeletal complications [5].

Sarcopenia and osteoporosis are among the late complications of T1D in musculoskeletal problems. Loss of muscle mass and function may occur earlier due to various factors in diabetes patients who are inclined to accelerate the aging process [6].

Therefore, there is a need to better understand the predictors of T1D-related musculoskeletal disease, so that effective clinical strategies can be developed, implemented and active life expectancy can be increased in T1D patients to prevent muscle loss, hence fractures and falling disease.

In our study, we aim to compare muscle strength and architecture between T1D patients and healthy volunteers and to determine whether there are structural changes in this population through ultrasonography.

## Methods

In the study, 32 T1D patients (23 female, 9 male) who admitted to the Gazi University, Faculty of Medicine applied to the Department of Endocrinology and Metabolism between April 2018 and April 2019 and 31 healthy (22 female, 9 male) volunteers paired in terms of age, gender, height, weight, physical activity were included. T1D patients were selected from follow-up patients diagnosed according to American Diabetes Association (ADA) criteria [7], while healthy volunteers were selected from hospital staff. Inclusion criteria were adults over the age of 18 followed for T1D, and exclusion criteria were; 1. Existence of a disease or drug use that affects the musculoskeletal system other than diabetes mellitus (Myopathy (Congenital, metabolic or acquired)), 2. History of neurological disease (stroke, multiple sclerosis, Parkinson's), 3. Radiculopathy, 4. Severe anemia, 5. Rheumatological disease, 6. A recent history of trauma, fractures, surgery, arthritis at the extremity to be measured, 7. Being engaged in professional sports, 8. Contraindications for isokinetic testing (Advanced heart failure, uncontrolled hypertension).

After the patients were verbally informed about the purpose and content of the study, the patients and volunteers who agreed to take part in the study were given written information and their support was obtained on the basis of the "Informed Consent Volunteer Form". Ethics committee approval was obtained for the study (Gazi University School of Medicine Ethics Committee No: 312, 31.04.2018) and the rules of the World Medical Association Declaration of Helsinki and the Guidelines for Good Clinical Practices were adhered to during the study process.

Initially, sociodemographic data of all participants, height, weight, body mass index, physical activity levels, dominant extremities and biochemical parameters (HbA1C, IGF-1, TSH, 25-OH vit D and cortisone) obtained during the follow-up process of the disease were recorded. HbA1c was measured using high-performance liquid chromatography (The G8 Variant Elution Buffer HSİ, Tosoh Corporation, Tokyo). Later, participants were evaluated for knee joint range of motion, deformities, joint laxity, sensitivity, swelling-heat increase and effusion. Detailed neurological examinations were performed for neuromuscular diseases with diabetic neuropathy assessment at the forefront. During neuropathy screening, neuropathic symptoms were questioned. Motor and sensory examinations (light touch, pinprick sense, joint position) were performed and the patient's gait pattern was observed. The sense of touch was evaluated with the 10 g monofilament test. Dominant lower extremity was evaluated by asking patients the preferred side when kicking a ball and standing on one foot. Retinopathy, nephropathy diagnoses of patients were determined verbally and by scanning file information.

### Evaluation of muscle strength

Muscle strength in the dominant knee in the patient and control group was measured using the Cybex 770 NORM isokinetic dynamometer system (Lumex Inc. Ronkonkoma, New York). Calibration was performed every time the device was opened before the evaluation process was carried out. All measurements were done by the same physician. Belts passing through the waist and thigh distal were used to ensure the stabilization of the participant.

During muscle strength measurements, the patient's knee was brought to full extension with the direction of the device and the anatomical 0 (zero) point was adjusted. In this position, joint ranges of motion openings were determined by bringing up to the highest extension/flexion angles that patients can make in the series. When setting the test protocol, the motion type was selected as concentric/concentric and the test was carried out at angular velocities of 60°/s and 180°/s. It was performed 5 times at 60°/s velocity and 20 times at 180°/s velocity. Before the tests carried out at both velocities, patients were given 4 re-trial tests to facilitate test adaptation. Patients were allowed to rest for 15–20 s between sets. All patients were allowed to see the power curve they revealed during the test on the screen, thus standardizing the visual feedback effect [8].

### Evaluation of muscle architecture

After the physical examination, taking into account that the participants will be tired during isokinetic testing, each volunteer was evaluated ultrasonographically on the same day before isokinetic tests by an expert in the field of musculoskeletal ultrasonography who was a blind observer to the clinical findings. Measurements of vastus lateralis (VL), vastus medialis (VM), vastus intermedius (VI) muscle thickness and pennate angles, rectus femoris (RF) muscle thickness were taken around the dominant knee.

**Fig. 1:**
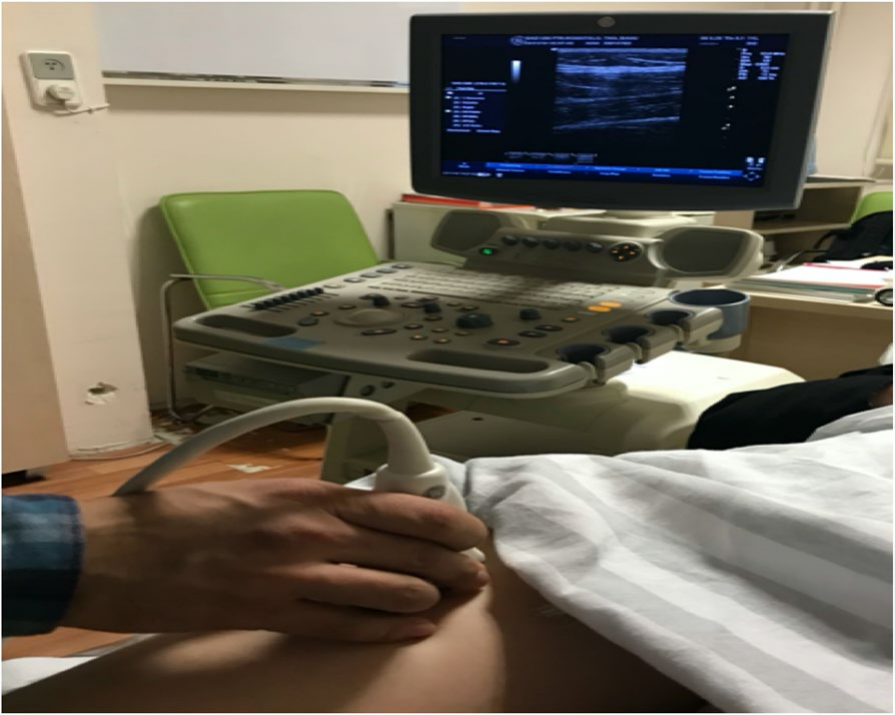
Rectus femoris—vastus intermedius ultrasonographic evaluation

**Fig. 2:**
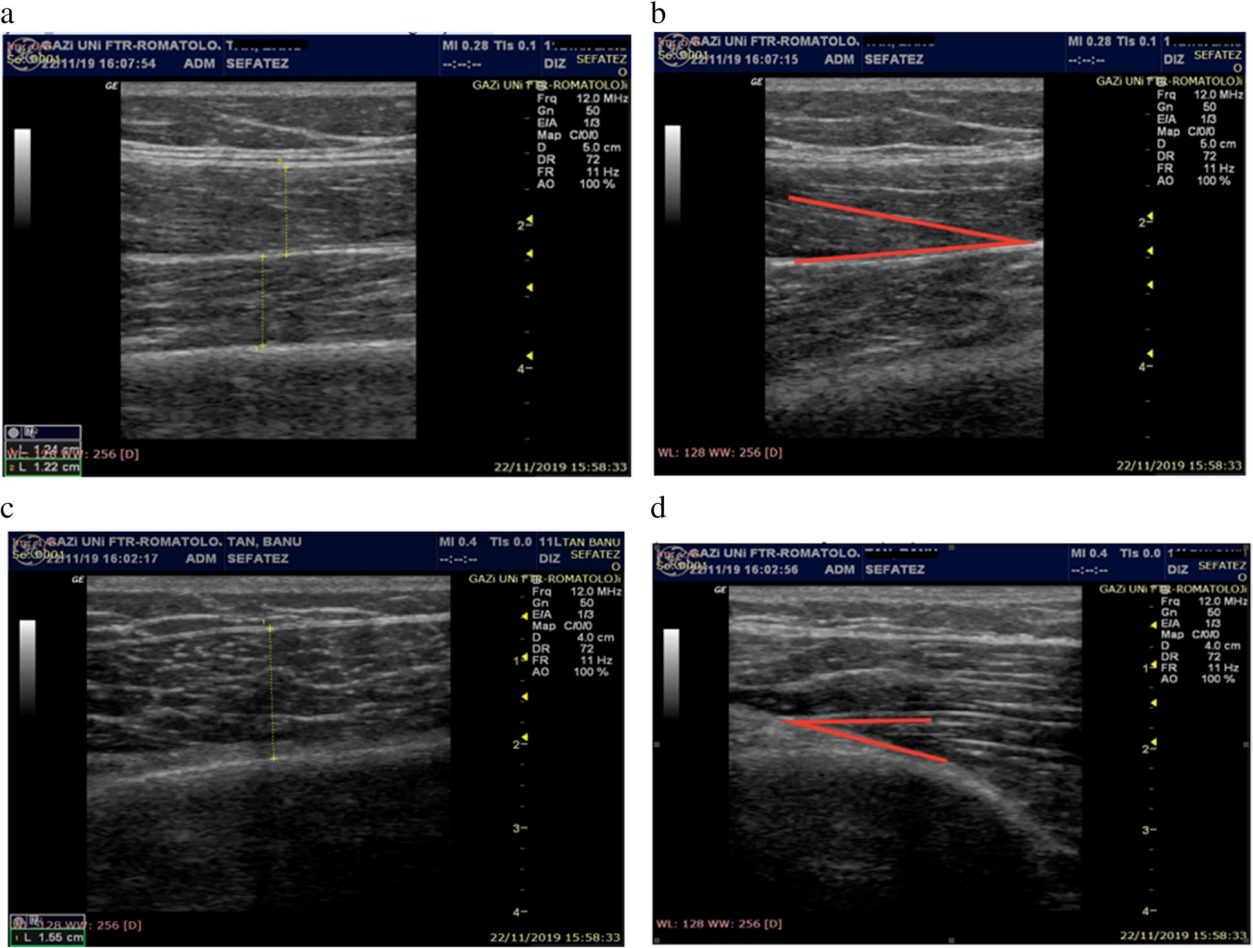
Muscle thickness* and pennate angle** measurements. **a** D1: Rectus femoris muscle thickness, D2: Vastus intermedius muscle thickness, **b** α: Rectus femoris pennate angle, **c** D3: Vastus medialis muscle thickness **d** α: Vastus medialis pennate angle *Muscle thickness: Distance between superficial and deep aponeurosis by holding the ultrasound probe axially and pressing it to the maximum, **Pennate angle: the angle formed at the attachment (with its insertion) of the muscle fascicles to the deep aponeurosis

MyLab 70 XV (Esaote Biomedica, Genoa, Italy) ultrasonography device, multifrequency linear probe and standard US gel were used for ultrasonographic evaluation. While patients are resting in the supin position, measuremets were taken for VM by marking the 3 cm superior- 4 cm medial of the femur medial condyle, for VL muscle by measuring the distance between spina iliaka anterior superior and femur lateral condyle and marking 2/3, and for RF and VI by measuring the distance between spina iliaka anterior superior and patella superior and marking ½ [9, 10]. Ultrasonographic evaluation is shown in Fig. 1, muscle thickness and pennate angle measurements are shown in Fig. 2.

### Evaluation of physical activity level

Since muscle strength may vary according to physical activity level, it was aimed to include individuals who were similar in physical activity to both groups. MET-min./week values indicating participants' level of physical activity and energy value of physical activity was determined by applying the 'International Physical Activity Quastionnare (IPAQ) Short Form'. IPAQ developed with the support of the World Health Organization (WHO) that also conducted its validity-reliability study [11].

### Statistical analysis

Statistical Package for Social Sciences (SPSS) version 22.0 (SPSS Inc. Chicago, USA) computer package program was used for statistical analysis of research data. The selected sample size was reached by power analysis. In the power analysis performed with 80% power and 5% margin of error to detect the possibility of 36 ± 37 Nm difference in the isokinetic outcome measure, the sample size was calculated as including 18 patients in each group [12]. In the descriptive statistics section, categorized variables were presented with numbers, percentages, and continuous variables with mean ± standard deviation and median (minimum–maximum). The normal distribution suitability of continuous variables was determined using visual (histogram and probability charts) and analytical methods (Kolmogorov–Smirnov/Shapiro–Wilk tests). As a result of normality analyses, independent sample t test was used for comparison analyses between the two groups if the data of continuous variables showed normal distribution and it the data did not Show normal distribution, Mann–Whitney U test was used. Chi-square test was used in comparison analyses for categorized variables between independent groups. The statistical significance level was accepted as *p* < 0,05 in the study.

## Results

Table 1 shows the demographic characteristics of the patients and control groups who were included in the study. There was no statistically significant difference between patient groups and control group in terms of physical activity level calculated by gender, age, height, weight, BMI, 25-hydroxy vitamin D levels and international physical activity survey (IPAQ) and met values indicating the energy value of physical activity (*p* > 0.05). The mean diabetes duration 15,46 year and HbA1C values 7,89 of the T1D group. In addition, 9 patients in the T1D group had hypothyroidism, all were using levothyroxine, the patients were at euthyroid level, and the mean levothyroxine dose was 77.5 mg.

**Table 1:** Demographic characteristics of the patient and control group

	**T1D Group**	**Control Group**	**Total**	***P* value(T1D-control)**
**Number of people**	32	31	63	
**Sex: F/M**	23/9	22/9	45/18	1
**Age (years)**				0,398^a^
Mean ± S	31.3 ± 8.7	33.3 ± 10.2	32.3 ± 9.5	
Median (Min – Max)	29.5(19–57)	30(21–59)	30(19–59)	
**Height (cm.)**				0.849^a^
Mean ± S	166.7 ± 8.98	166.2 ± 11.3	166.5 ± 10.1	
Median (Min – Max)	165(152–191)	165(150–190)	150–191	
**Wieght (kg.)**				0.483^a^
Mean ± S	66.2 ± 10.3	68.1 ± 11.3	67.1 ± 10.7	
Median (Min – Max)	67.5(45–84)	63(53–88)	45–88	
**BMI (kg/m**^**2**^**)**				0.279^a^
Mean ± S	23.7 ± 3.1	24.6 ± 3.3	24.2 ± 3.2	
Median (Min – Max)	23.9(18.1–29)	24.24(18.7–33.3)	18.1–33.3	
**IPAQ**				0.632^b^
Mean ± S	1610.1 ± 1907.9	1557.8 ± 1537.8	1586.85 ± 1737.1	
Median (Min – Max)	693(165–7092)	973.5(172–5544)	716.5(165–7092)	
**25(OH) vit D (ng/ml)**				0.383^a^
Mean ± S	18.3 ± 5.9	14.3 ± 5.6	16.3 ± 5.8	
Median (Min – Max)	18.5(7–33)	14.1(5–27)	16.3(5–33)	

*S* Standart deviation, *Min* Minimum, *Max* Maximum, *BMI* Body mass index, *IPAQ* International Physical Activity Questionnaire, *25(OH) vit D* 25-hydroxy vitamin D

^**a**^Independent Sample t-test, ^b^Mann Whitney U test

Isokinetic test measurements of both groups are given in Table 2. At an angular velocity of 60°/s, there was a statistically significant difference between the T1D group and the control group in terms of isokinetic peak torque values of the hamstring and quadriceps muscles (flexion and extension) (*p* = 0.011, *p* = 0.015, respectively). Peak torque values at angular velocity of 180°/s were lower in the T1D group, but this difference was not statistically significant (*p* = 0.277, *p* = 0.319, respectively).

**Table 2:** Comparison of isokinetic test parameters of patient and control group

	T1D Group	Control Group	*p* value
60° flexor peak torque (Nm)			**0.011**^**a**^
Mean ± S	38.2 ± 14.8	53.7 ± 25.6	
Median (Min – Max)	34(14 – 72)	50(23 –137)	
60° extensor peak torque (Nm)			**0.015**^**a**^
Mean ± S	69.5 ± 28.5	91.4 ± 28.9	
Median (Min – Max)	22(23 – 137)	31(27 – 213)	
180° flexor peak torque (Nm)			0.277^a^
Mean ± S	24.5 ± 11.9	29.6 ± 17	
Median (Min – Max)	22(9 – 56)	31(8– 81)	
180° extensor peak torque (Nm)			0.319^a^
Mean ± S	31.4 ± 17.7	39.7 ± 26.9	
Median (Min – Max)	27.5(9 – 83)	33(8–119)	

*S* Standart deviation, *Min* Minimum, *Max* Maximum, *Nm* Newton-meter

^a^Mann Whitney U test

When the muscle architecture measurements of both groups were considered (RF, VI, VM, VL muscle thickness and VI, VM, VL pennate angle measurements), there was a significant difference in RF, VI, VM, VL muscle thicknesses and VM, VI pennate angle measurements in the T1D group compared to the control group. Muscle architecture measurements are given in Table 3.

**Table 3:** Comparison of muscle thickness and pennate angle measurements of patients and control groups

	T1D Group	Control Group	*p* value
Rectus femoris Muscle thickness			0.013^a^
Mean ± S	1.8 ± 0.3	1.98 ± 0.26	
Median (Min – Max)	1.84(1.09 –2.5)	1.93(1.57– 2.72)	
Vastus intermedius Muscle thickness			0.047^a^
Mean ± S	1.73 ± 0.35	1.92 ± 0.42	
Median (Min – Max)	1.80(0.9 – 2.48)	1.89(0.99 – 2.79)	
Vastus intermedius Pennate angle			< 0.001^b^
Mean ± S	13.19 ± 4.26	17 ± 3.95	
Median (Min – Max)	13(6 – 27)	17(10–24)	
Vastus medialis Muscle thickness			0.011^b^
Mean ± S	1.94 ± 0.32	2.19 ± 0.43	
Median (Min – Max)	1.96(1.38–2.63)	2.1(1.56 – 3.88)	
Vastus medialis Pennate angle			< 0.001^b^
Mean ± S	17.56 ± 7.2	25.18 ± 7.34	
Median (Min – Max)	15.5(7–3)3	27(8–36)	
Vastus lateralis Muscle thickness			0.043^a^
Mean ± S	1.58 ± 0.27	2.73 ± 0.31	
Median (Min – Max)	1.57(0.85 – 2.3)	1.75(1.22 – 2.5)	
Vastus lateralis Pennate angle			0.200^a^
Median	11.93 ± 3.12	13.09 ± 3.95	
Median (Min – Max)	12(7–18)	13(7–23)	

*S* Standart deviation, *Min* Minimum, *Max* Maximum

^a^Independent Sample t-test.^b^Mann Whitney U test

According to the method of administering insulin (subcutaneous injection, insulin pump), 18 patients (56%) used insulin pumps and 14 patients (44%) received subcutaneous insulin therapy. There was no significant difference between the two groups for age, height, weight, body mass index, total insulin dose and level of physical activity. Demographic data based on the method of using insulin are given in Table 4.

**Table 4:** Demographic characteristics of subgroups according to insulin application method

	**Insulin pump**	**Subcutan** **Insulin**	***p* value**
**Number of people**	18	14	
**Sex: W/M**	12/6	11/3	0.694^a^
**Age (year)**			0.543^b^
Mean ± S	32.3 ± 9	30 ± 8.54	
Median (Min – Max)	29.5(20–57)	30(19–44)	
**Height**			0.331^b^
Mean ± S	168.38 ± 9.6	164.64 ± 7.8	
Median (Min – Max)	166(157–191)	163(152–176)	
**Weight**			0.098^b^
Mean ± S	69.11 ± 7.2	62.46 ± 12.7	
Median (Min – Max)	68(51–84)	62.5(45–84)	
**BMI (kg/m**^**2**^**)**			0.210^b^
Mean ± S	24.4 ± 2.78	22.87 ± 3.38	
Median (Min – Max)	24(20.06–29)	22.1(18.1–28.9)	
**IPAQ**			0.769^b^
Mean ± S	1701.65	1490.31	
Median (Min – Max)	693(198–7092)	594(165–5439)	
**Insulin Dose**			0.621^**b**^
Mean ± S	41.72 ± 12.3	40 ± 15.08	
Median (Min – Max)	43.5(18–58)	36(23–72)	

*S* Standart deviation, *Min* Minimum, *Max* Maximum, *BMI* Body mass index, *IPAQ* International Physical Activity Questionnaire

^a^Ki-kare test.^b^Mann Whitney U test

Compared to the insulin application method, in the group using insulin pump, muscle strength values were higher but not statistically significant in all measurements (60 flexor peak torque, 60 extensor peak torque, 180 flexor peak torque, 180 extensor peak torque) compared to the group that received subcutaneous insulin therapy. In Table 5, isokinetic test parameter results were shown according to the method of using insulin.

**Table 5:** Isokinetic test parameters according to the method of using insulin in the patient group

	**Insulin pump**	**Subcutan** **Insulin**	***p* value**
60° Flexor peak torque (Nm)			0.128^a^
Mean ± S	41.33 ± 16.05	34.21 ± 12.49	
Median (Min – Max)	39(14–72)	30.5(22–65)	
60° Extensor peak torque (Nm)			0.392^a^
Mean ± S	66.61 ± 29.16	73.35 ± 28.29	
Median (Min – Max)	60(23–137)	77.5(23–117)	
180° Flexor peak torque (Nm)			0.132^a^
Mean ± S	27.77 ± 13.73	20.14 ± 7.73	
Median (Min – Max)	27(9–56)	17.5(9–33)	
180° Extensor peak torque (Nm)			0.761^a^
Mean ± S	32.27 ± 18.99	30.42–16.53	
Median (Min – Max)	28(9–83)	26.5(9–60)	

*Min* Minimum, *Max* Maximum, *Nm* Newton-meter

^a^Mann Whitney U test

When the ultrasonographic measurement parameters according to insulin application method were compared in the patient group, muscle thickness and pennate angle measurements were higher in all measurements, and RF muscle thickness (*p* = 0.11), VI muscle thickness (*p* = 0.038), VM muscle thickness (*p* = 0.004) and VI angle (*p* = 0.069) measurements were statistically significant according to the group that received subcutaneous insulin treatment. In Table 6, muscle architecture measurement results are shown according to insulin application method.

**Table 6:** Ultrasonographic measurement parameters according to insulin application method in the patient group

	Insulin Pump	Subcutan Insulin	*p* value
Rectus femoris Muscle thickness			0.011^a^
Mean ± S	1.91 ± 0.2	1.66 ± 0.32	
Median (Min – Max)	1.9(1.56–2.5)	1.7(1.09–2.31)	
Vastus intermedius Muscle thickness			0.038^a^
Mean ± S	1.93 ± 0.31	1.61 ± 0.41	
Median (Min – Max)	1.92(1.31–2.48)	1.65(0.9–2.3)	
Vastus intermedius Pennate angle			0.069^a^
Mean ± S	14.47 ± 4.5	11.64 ± 3.49	
Median (Min – Max)	14(8–27)	11(6–18)	
Vastus medialis Muscle thickness			0.004^a^
Mean ± S	2.07 ± 0.24	1.77 ± 0.35	
Median (Min – Max)	2(1.78–2.63)	1.71(1.38–2.62)	
Vastus medialis Pennate angle			0.323^a^
Mean ± S	18.88 ± 7.36	15.85 ± 6.87	
Median (Min – Max)	15(10–33)	16.5(7–31)	
Vastus lateralis Muscle thickness			0.254^a^
Mean ± S	1.62 ± 0.21	1.53 ± 0.34	
Median (Min – Max)	1.65(1.15–1.95)	1.52(0.85–2.3)	
Vastus lateralis Pennate angle			0.181^a^
Mean ± S	12.66 ± 3.42	11 ± 2.48	
Median (Min – Max)	12.5(7–18)	11(8–15)	

*Min* Minimum, *Max* Maximum

^a^Independent Sample t-test

## Discussion

To the best of our knowledge, this study is the first study in the literature comparing isokinetic test parameters and muscle architecture measurements in healthy volunteers taken as control group and T1D. The relationship between knee flexion and extension peak torque measurements (60°-180°) and quadriceps femoris muscle thicknesses and pennate angles were measured separately and the relationship between various demographic and clinical parameters was examined. According to the literature, studies in the field of diabetes mellitus and muscle strength were carried out mainly in T2D patients. Similar to our study, in addition to studies reporting a decrease in muscle strength in the diabetic population [12, 13], there are also studies showing that diabetes does not cause a significant difference in muscle strength [14, 15].

Sacchetti et al. [12] compared the isometric and isokinetic measurements of elbow flexor and knee extensor muscle groups at different angular velocities in sedentary and trained diabetic patients classified according to motor nerve impairment and in the control group. There was no difference in isometric measurements of both elbow flexors and knee extensors. In the isokinetic measurements, a significant decrease was found in the peak torque values ​​at high angular velocities in the elbow flexors, and a decrease in the peak torque values ​​at all angular velocities in the knee extensors in the diabetic group. We also performed the measurements in our study in the knee joint, since the isokinetic measurement results in the knee joint are more consistent than the other measurement results in our clinical practice. Unlike our study, the reason for the decrease in all angular velocities in knee extensor isokinetic measurements may be because of motor nerve impairment in the diabetic group. In addition, the fact that the study was predominantly conducted in the type 2 diabetes group (only 8 T1D), the diabetes group was older (mean age 60 ± 10.7) and the diabetes duration was long (21.4 ± 8.2) may be the reason for the significant decrease in all angular velocities. These changes in muscle strength depending on the contraction velocity in diabetics show that it is important to test at different angular velocities. In the future this topic, prospective cohort studies can be carried out and exercise training models specific to diabetics can be developed according to the data to be obtained.

Many factors have been shown to play a role in the development of diabetic myopathy, such as hyperglycemia and increased polyol activation caused by it, protein glycation (AGE), decreased insulin by atrogene expression as a result, impaired GH/IGF-1 axle, increased glycocorticoid, IL-6, PAI-1, and changes in key hormones and cytokines [16]. As a result, an increase in muscle proteolysis, a decrease in protein synthesis and atrophy of glycolytic muscle fibers may occur. Histopathological studies in T1D also show atrophy unique to muscle fiber type. Accordingly, there was a minimum loss or slight increase in type 1 fibers, while heavy atrophy was detected in type 2B fibers [17].

In our study, peak torque values obtained at different angular velocities may be consistent with histopathological changes occurring in T1D muscle cells. In our study, flexion and extension peak torque values were significantly lower in the T1D group at angular velocity of 60 º/s, where fast contracting and fast- tired type 2D fibers were active. The decrease in flexion and extension values at angular velocity of 180 º/s, where type 1 fibers responsible for fatigue-resistant endurance were more responsible, was not statistically significant.

In muscle architecture measurements, there was a significant decrease in RF, VI, VM, VL muscle thickness measurements and VM, VI pennate angle measurements in T1Ds, and VL pennate angle measurement was not significant. This decrease in the pennate angle values previously shown to be in correlation with muscle strength [18] also supported other results. These values reveal that there may be significant changes in muscle architecture due to the absence of insulin and this can be shown ultrasonographically.

The reason for the lack of significant changes in VL muscle in T1D patients can be explained by the wide fiber variation of the muscle. While it is known that the muscles in which tonic activity is dominant contain highly type 1 fibers, while the muscles where phasic activity is dominant are known to contain a higher proportion of type 2 fibers, interestingly, Brooke and Engel studies have shown a wider variation in the biceps brakii and vastus lateralis muscles [19].

Muscle architecture measurements (RF, VI, VM muscle thickness measurements) were lower in the group using subcutaneous insulin. In the group using insulin pump, the reason for better measurements in muscle strength and architecture may be associated with the superiority of insulin pump in intraday glycemic variability [20].

When the muscle strength measurements of the patient population in our study was compared according to HbA1c values (HbA1C < 8, HbA1C > 8), there was no significant difference in isokinetic test parameters between the two groups. This is because the number of patients falling into groups in our sub-group analyses may be less than the number of people we identified in our power analysis at the beginning of our study. We also thought that the HbA1C values obtained from patients may not have changed muscle strength measurements due to the pre-procedure values and the lack of an indication of long-term monitoring of the disease in terms of glycemic control (Data not shown).

Furthermore, some studies show that poor glycemic control in people with T1D is associated with both micro and macrovascular complications, but good glycemic control does not fully prevent the risk of these complications. Common metabolomic abnormalities were identified with poor glycemic control in patients with T1D, but most of these deteriorations detected in those with poor glycemic control were shown to remain unchanged in those with chronically good glycemic control [21]. Similarly, there was no significant difference in muscle strength and architecture in those with better glycemic control.

It's hard to tell whether the muscle abnormalities are related to diabetes alone or to other factors (diabetic micro/macrovascular complications). This is one of the factors that directs us to work on this subject, but also limits the study. However recently, there have been studies showing that muscle strength deterioration develops independently and before neuropathy [22, 23]. The fact that these observations occur regardless of the presence of diabetic neuropathy is associated with the sensitivity of the skeletal muscle to the T1D environment.

The skeletal muscle is the largest insulin-sensitive organ. Given its contribution to the control of glycemic, it can be speculated that deterioration in skeletal muscle health in T1D may be the primary factor in the progression of other complications of diabetes. Data from human studies highlight significant functional (loss of strength generation, increased fatigue, loss of muscle stem cells) and mitochondrial (metabolic) dysfunctions that may affect the ability to alleviate dysglycemia and dyslipidemia in muscles [24]. Therefore, the development of diabetic myopathy not only leads to weakness and increased fatigue in the muscles, but may also exacerbate the development and progression of other diabetic complications due to functional and mitochondrial dysfunction in the skeleton muscle – decreased physical activity capacity, impaired substrate oxidation and decreased insulin sensitivity [25]. In other words, good musculoskeletal health can also be important in slowing the progression of T1D and other complications.

The strengths of our study are that ultrasonographic and isokinetic evaluation was carried out simultaneously, that it was the first study in the literature where muscle architecture was evaluated in T1D patients, and that it was leading future studies on the model that needs to be developed to understand diabetes myopathy and underlying mechanisms.

There are also some limitations of our study. These can be listed as the lack of sufficient number of patients for propensity matching in subgroup analysis, the lack of electrophysiological study for the definitive diagnosis of diabetic neuropathy and testosterone levels that may lead to sarcopenia were not done in males. Additionally, primary hypothyroidism may be associated with changes in muscle architecture and strength. Therefore, in this study, hypothyroidism can be mentioned as a confounding factor, even though the patients are at euthyroid level.

Evidence to date suggests that diabetic myopathy as a neuromuscular disorder should be considered as one of the major complications of diabetes. The relationship between skeletal muscle health and general well-being is better known nowadays [26]. Especially in the T1D group, the importance of skeletal muscle and health is better understood. Identifying the extent of myopathy in patients with T1D will reveal the mechanical perspective of the adaptive capacities of T1D muscles and may help evidence-based therapeutic exercise strategies to improve the functional and metabolic capacities of skeletal muscle in T1D.

In conclusion, muscle strength and architecture were adversely affected in patients with T1D and diabetic myopathy developed independently of other diabetic complications. Therefore, it is important and necessary to start therapeutic exercises from the early stages of diabetes in order to better achieve glycemic control in the T1D group, to prevent the progression of other complications and to increase the active life expectancy of patients.

## Data Availability

The datasets used and/or analysed during the current study available from the corresponding author on reasonable request.

## Abbreviations

AGE: Advanced glycation end product
GH: Growth Hormone
IGF-1: Insulin like growth factor-1
IPAQ: International Physical Activity Questionnaire
RF: Rectus femoris
T1D: Type 1 diabetes mellitus
T2D: Type 2 diabetes mellitus
USG: Ultrasonography
VI: Vastus interedius
VL: Vastus lateralis
VM: Vastus medialis
